# Symbiont-Mediated Insecticide Detoxification as an Emerging Problem in Insect Pests

**DOI:** 10.3389/fmicb.2020.547108

**Published:** 2020-09-30

**Authors:** Alison G. Blanton, Brittany F. Peterson

**Affiliations:** Department of Biological Sciences, Southern Illinois University Edwardsville, Edwardsville, IL, United States

**Keywords:** symbiosis, microbiota, insecticide detoxification, symbiont-mediated, host-microbe interactions

## Abstract

Pesticide use is prevalent with applications from the backyard gardener to large-scale agriculture and combatting pests in homes and industrial settings. Alongside the need to control unwanted pests comes the selective pressure generated by sustained pesticide use has become a concern leading to environmental contamination, pest resistance, and, thus, reduced pesticide efficacy. Despite efforts to improve the environmental impact and reduce off-target effects, chemical pesticides are relied on and control failures are costly. Though pesticide resistance mechanisms vary, one pattern that has recently emerged is symbiont-mediated detoxification within insect pests. The localization within the insect host, the identity of the symbiotic partner, and the stability of the associations across different systems vary. The diversity of insects and ecological settings linked to this phenomenon are broad. In this mini-review, we summarize the recent trend of insecticide detoxification modulated by symbiotic associations between bacteria and insects, as well as highlight the implications for pesticide development, pest management strategies, and pesticide bioremediation.

## Introduction

Environmental consciousness has increased awareness of pesticide use patterns, environmental stability, and off-target effects. This has resulted in consumer demands and policy implementation geared toward environmental sustainability including a push for green chemistries, biological control, and bioremediation (Tilman et al., 2002; Damalas and Koutroubas, 2018; Thomas et al., 2019). Appreciation for microbial transformation of toxins, pollutants, and pesticides has only increased with these campaigns pushing for the adoption of ecological pest management strategies (Bozkurt, 2017; Abrol and Shankar, 2019). Bacteria have been found to detoxify multiple insecticides classes including pyrethroids, neonicotinoids, and organophosphates (Sethunathan and Yoshida, 1973; Fisher et al., 1978; Serdar and Gibson, 1985; Chaudhry and Huang, 1988; Bhat et al., 1994; Nadeau et al., 1994; Hayatsu et al., 2000; Kamal et al., 2008; Boricha and Fulekar, 2009; Zhang et al., 2011; Kong et al., 2013; Nayarisseri et al., 2015; Pankaj et al., 2016; Shetti and Kaliwal, 2016; Fernández-López et al., 2017; Gangola et al., 2018; Kumar, 2018; Aswathi et al., 2019; Meng et al., 2019).

Though bacterial pesticide degradation research has been steady over the past 50 years, understanding the association of these bacteria with insects has been limited until recently. While insecticide biotransformation by endogenous means is well documented in insects (Panini et al., 2016; Bass and Jones, 2018), bacterial symbionts as mechanism for *in insecta* insecticide detoxification was not documented until the mid-twentieth century. In 1967, organophosphate detoxification by a bacterial symbiont of the apple maggot was reported (Boush and Matsumura, 1967). This study demonstrated degradation of six different insecticide active ingredients by an insect-associated bacterium, but the significance of this finding to the insect host and whether it conferred pesticide tolerance remained unknown.

This idea, bacterial degradation of insecticides connected with an insect host, was revitalized decades later when *Burkholderia* colonizing bean bug midguts were demonstrated to confer resistance to the organophosphate pesticide fenitrothion to their insect hosts (Kikuchi et al., 2012). This study reignited interest in symbiont-mediated pesticide detoxification and introduced facultative associations with environmentally acquired bacteria as a mechanism for pesticide tolerance.

In this mini-review, we focus on documenting instances of symbiont-mediated insecticide detoxification within host insects and discussing the implications of this phenomenon. The repeated discovery of both facultative and obligate pesticide detoxifying symbioses across insect taxa emphasizes the need for more research in this area. Importantly, we largely focus on insect gut symbionts that encounter insecticides through host ingestion, but it is important to note that this distinction may be unnecessary given data regarding symbiont mitigation of topically applied pesticides (Kikuchi et al., 2012). Additionally, insect-associated bacteria capable of detoxifying pesticides will be important to consider in the design of future, novel active ingredients to ensure their long-term efficacy. Finally, as we continue to seek creative ways to mitigate the environmental impacts of pesticide use, perhaps we look no further than the bacteria in the very pests and fields targeted for an opportunity for bioremediation.

## Insect Symbionts Extend and Expand Host Physiological Capabilities

Bacterial symbionts have been found to perform many complex metabolic processes within their host insects (Su et al., 2013). In insects, bacterial symbionts are known to confer protection against antagonists (Oliver et al., 2003; Kaltenpoth et al., 2005; Parker et al., 2013), digest or supplement suboptimal diets (Gunduz and Douglas, 2009; Sabree et al., 2009; Peterson et al., 2015), and play critical roles in development (Dedeine et al., 2001; Kafil et al., 2013; Lee et al., 2017). In addition to these examples, symbionts have been implied or inferred to play roles in many other processes.

The relationships between insect hosts, their symbionts, and pesticides have been alluded to in the literature. Termites treated with a neonicotinoid insecticide are more susceptible to pathogens (Sen et al., 2015). In honey bees, the composition and abundance of gut bacteria have been linked to pesticide exposure (Kakumanu et al., 2016; Motta et al., 2018). However, these examples aim to understand the detrimental impacts of pesticides on insect microbiota rather than implicating those bacteria in the degradation of pesticides. Along those lines, herbivore-associated bacteria have long been credited with neutralizing the toxic metabolites of plants (reviewed in van den Bosch and Welte, 2017; Giron et al., 2017; Itoh et al., 2018) and linked to modulation of plant defense systems in favor of their insect hosts (Chung et al., 2013). So, while perhaps unsurprising, there is a growing number of studies that directly link insect symbionts to the detoxification of insecticides and/or confer tolerance to insecticides to their hosts.

### Discovery of a Novel Role for Symbionts in Insect Hosts

*Rhagoletis pomonella*, the apple maggot, larvae develop in the fruit of apple trees causing significant damage. During the mid-twentieth century, organophosphate pesticide use in agriculture was prevalent (Croft, 1982). Though reports in the late 1970s and early 1980s suggested no widespread organophosphate resistance in apple pests (Croft, 1979; Croft, 1982), one study investigated potential detoxification mechanisms of organophosphate pesticide in *R. pomonella* (Boush and Matsumura, 1967). *R. pomonella* is associated with the bacterium *Pseudomonas melophthora* (Allen et al., 1934). *P. melophthora* has been found both in the gut of the apple maggot and in the surrounding soft rot of the fruit where apple maggots have taken up residence. At the time, this association between the symbiont and *R. pomonella* was attributed to the role of *P. melophthora* as a plant pathogen (Boush and Matsumura, 1967). However, after culturing and identifying the bacterial symbiont, the authors provided evidence that *P. melophthora* could degrade organophosphates and speculated bacterial esterases as the degradation mechanism (Boush and Matsumura, 1967).

While perhaps not as appreciated then, this study was the first to suggest a novel role in pesticide detoxification for symbionts within insects (Boush and Matsumura, 1967). Though the direct impact these symbionts would have on host insect resistance remained unclear. Likewise, whether the action of these bacterial esterases were selected specifically for organophosphate degrading activity or if they were simply generic detoxification enzymes recruited for this activity is not known. Following this study, no additional direct evidence of symbiont-mediated pesticide interactions within-insects would emerge for 45 years. Interestingly, the mechanism elucidated by Boush and Matsumura in the 1960s, bacterial esterase biotransformation of chemical insecticide, has been found several subsequent studies identifying bacterial mechanisms of insecticide degradation (Boush and Matsumura, 1967; Fisher et al., 1978; Kamal et al., 2008; Gangola et al., 2018; Table 1).

**TABLE 1:** Summary of insect species, their associated bacteria, pesticides degraded by the symbionts, and the proposed detoxification mechanism utilized.

**Insect host**	**Symbiont**	**Pesticide(s)**	**Detox mechanism(s)**	**References**
*Rhagoletis pomonella*	*Pseudomonas melophthora*	Dichlorovos Diazinon Parathion Diisopropyl phosphorofluoridate Dieldrin Carbaryl	Esterase (hypothesized)	Boush and Matsumura, 1967
*Lasioderma serricorne*	*Symbiotaphrina kochii*	Parathion	Aryl-ester hydrolase Glucosidase Phosphatase Glutathione transferase	Shen and Dowd, 1991
*Culex pipiens*	*Wolbachia*	Organophosphates*	Not specified	Berticat et al., 2002
*Riptortus pedestris & Cavelerius saccharivous*	*Burkholderia* sp.	Fenitrothion Diazinon EPN Isoxathion	Not Specified	Kikuchi et al., 2012
*Spodoptera frugiperda*	Gut microbiota*	Deltamethrin λ-Cyhalothrin Chlorpyrifos Spinosad Lufenuron	Monooxygenase Esterase Hydrolase Transferase	de Almeida et al., 2017
*Anopheles stephensi*	*Pseudomonas Aeromonas Exiguobacterium Microbacterium*	Temephos	α-Esterase Glutathione S-transferase Acetylcholine esterase	Soltani et al., 2017
*Bactrocera dorsalis*	*Citrobacter* sp.	Trichlorphon	Phosphatase	Cheng et al., 2017
*Plutella xylostella*	*Enterococcus* sp.	Chlorpyrifos	Not specified	Xie et al., 2018
*Blatella germanica*	Gut microbiota*	Indoxacarb	Not specified	Pietri et al., 2018
*Nilaparvata lugens*	*Arsenphonus*	Imidacloprid Buprofezin	Not specified	Pang et al., 2018
*Callosobruchus maculates*	Gut microbiota*	Dichlorvos	Not specified	Akami et al., 2019

*Indicates details of specific taxa or pesticides are not known or not explicitly stated.

### Bean Bugs and *Burkholderia* Reignite the Study of Symbiont-Mediated Pesticide Detoxification

The burgeoning field of insect-symbiont associations at the turn of the century was facilitated with the advancement of culture-independent technologies. As mentioned above, the appreciation for bacteria-mediated physiological capabilities in association with and in complementarity to insect hosts exploded in the last 20 years. Perhaps it was only a matter of time before the observation of Boush and Matsumura was revisited. In 2012, a population of bean bugs, *Riptortus pedestris*, were suddenly resistant to fenitrothion within a single field season. Subsequent investigation revealed a correlation between particular *Burkholderia* symbionts and resistant *R. pedestris* individuals (Kikuchi et al., 2012). *Burkholderia* symbionts are known associates of true bugs (Kikuchi et al., 2007, 2011). These bacteria are harbored in specialized regions of the midget and are link to proper growth and development of the insect (Kikuchi et al., 2007, 2011). Perhaps most importantly, host insects could acquire these *Burkholderia* symbionts directly from the soil and by harboring them in the crypts of their digestive tract, tolerate both fenitrothion ingestion and topical application. Fenitrothion treatment enriched soil for degradation activity and another species, *Cavelerius saccharivous*, collected from fields regularly sprayed with fenitrothion are also associated with degrading strains of *Burkholderia.* From this, authors asserted that environmental pressure plays a large role in the acquisition of pesticide degrading *Burkholderia* symbionts by true bugs, like *R. pedestris* and *C. saccharivorous* (Kikuchi et al., 2012).

### Patterns of Symbiont-Mediated Pesticide Detoxification Emerge Across Insect Taxa

Use of chemical pesticides has long been associated with the development of resistance, but that resistance to a chemical insecticide could sweep through a population within a single generation, as was observed in bean bugs, is alarming (Kikuchi et al., 2012). This was striking, because a facultative, environmentally-acquired symbiont being link to such a fast-moving phenotype was unprecedented. These *Burkholderia* symbionts were also found to degrade other organophosphate pesticides suggesting a possible broader impact on insecticide resistance within host insects (Kikuchi et al., 2012). Though previous papers had shown interplay between insect microbiomes and natural enemies, like *Bacillus thuringiensis*, this mechanism for pesticide tolerance had not been observed since Boush and Matsumura (Boush and Matsumura, 1967; Oliver et al., 2003; Broderick et al., 2006; Peterson and Scharf, 2016).

Subsequent studies and their resulting insights highlight the complexity and extent of insect-microbe collaboration regarding pesticide tolerance and biotransformation. These findings, their caveats and insights, and some perspectives on their impact on biotechnology were reviewed recently (Pietri and Liang, 2018). Briefly, *Lasioderma serricorne, Culex pipiens, Anopheles stephensi*, *Bactrocera dorsalis, Plutella xylostella, Spodoptera frugiperda, Nilaparvata lugens, Blatella germanica*, and *Callosobruchus maculatus* have all been found in association with bacteria mediate or modulate increased pesticide tolerance (Shen and Dowd, 1991; Berticat et al., 2002; Soltani et al., 2017; Cheng et al., 2017; de Almeida et al., 2017; Xie et al., 2018; Pang et al., 2018; Pietri et al., 2018; Akami et al., 2019; Table 1). Using reductive antibiotic treatment approaches and *in vitro*, culturing assays many of these studies directly link the presence and/or metabolic capabilities of symbiotic microbes to pesticide detoxification abilities (Shen and Dowd, 1991; de Almeida et al., 2017; Cheng et al., 2017; Soltani et al., 2017; Pietri et al., 2018; Xie et al., 2018; Akami et al., 2019; Table 1). And, similar to the example in *R. pedestris*, specific *Arsenophonus* strains have being linked to pesticide susceptibility in a leafhopper species (Pang et al., 2018). These examples come from diverse insect groups with distinct life histories and ecological interactions. This reemphasizes that this emerging phenomenon is not restricted to any particular taxon or niche. This discovery renewed interest in the interactions of insect symbionts with chemical pesticide and provided context for a new perspective on insecticide resistance and pesticide development.

## Implications for Insecticide Development, Pest Control, and Bioremediation

The diverse examples of pesticide degradation via insect symbionts (Table 1) have many applications, particularly for the futures of pest control and bioremediation. Pest control is a dynamic arms race: on one side farmers, pesticide developers, agribusiness, and scientists all work to maintain crop protection and encourage production (Damalas and Koutroubas, 2018) and on the other side insects and other pests follow their biological drive to live and reproduce. With the intense selective pressures on pest species with heavy insecticide use, we see the potential for control failures, cross-resistance, and off-target impacts. Using the knowledge of symbiont-mediated detoxification, there is potential for the development of symbiont targeted pesticides which exploit the interplay of symbiont-host interactions. This concept has foundations in our understanding of insect gut microbiota as it relates to pro-insecticide metabolism (Daisley et al., 2018) and presents another opportunity to synergize our understanding of insect-bacteria associations with our need to control pest populations. Particularly, leveraging known mechanisms of bacterial colonization and adherence within hosts could be important for the development of active ingredients rather than deploying antibiotics into the environment. Development of such pesticides would be relevant across a variety of environments as well both urban and agricultural due to the variety of hosts associated with such bacteria (Broderick et al., 2006; van den Bosch and Welte, 2017; Pietri and Liang, 2018). In addition to exploiting knowledge of these insect-microbe symbioses as control targets, when disassociated, these bacteria may be useful for the restoration of environments contaminated with out-of-use active ingredients.

### Potential Strategies for Using Insect-Associated Bacteria for Bioremediation

Using bioremediation developed for oil and other environmental contaminants as a guide, we can begin to explore how insect-associated bacteria may be deployed for environmental clean-up. The addition of either one particular bacterium or a bacterial consortium to soil or waterways reduces the half-life of these contaminants (Vasudevan and Rajaram, 2001; Singh et al., 2004; Gentili et al., 2006; Shivaramaiah and Kennedy, 2006). Importantly, the source of these bacteria are often indigenous to contaminated environments. The practice of bioaugmentation, that is increasing the density and abundance of bacteria capable of bioremediating environmental contaminants, has been successful with oil-degrading species like *Alcanivorax burkumensis* (Hassanshahian et al., 2014; Kadri et al., 2018). Using these practices as a guide, insect-associated, insecticide-degrading bacteria are advantageous because they are linked with the use of the active ingredient. Partnered with genetic engineering and large-scale production, deployment of bacterial symbionts from insects may be well-suited for bioaugmentation efforts in areas where defunct active ingredients linger (Pieper and Reineke, 2000). This would allow for remediation of areas such as agricultural fields or restored areas with a history of pesticide utilization. This may be particularly useful for the environmental cleanup of products where further use has been banned or ecological impacts persist (like off-target effects or bioaccumulation).

Any *in situ* bioaugmentation or inoculation to control environmental contaminants should be coupled with a monitoring program (Naik and Dubey, 2013). To monitor for the presence of pesticide degrading bacteria, diagnostic PCR must be developed and regular testing of soil, water, and potential host insects could be implemented (Kohno et al., 2002; Kikuchi et al., 2012). To mitigate the concerns of horizontal gene transfer, these methods of bioremediation would be utilized under circumstances where a pesticide has been discontinued. Additionally, if utilizing bioengineered or optimized bacteria there may be potential to include an exclusion system to prevent subsequent conjugation events and limit lateral gene transfer to other bacteria in the environment (Avello et al., 2019).

### Limitations and Areas of Need for the Deployment of Insect-Associated Bacteria for Bioremediation

While the potential impact these symbiotic microbes have is great, it is important to recognize the current limitations of this concept. Chiefly, the research in this area is ongoing and extremely limited. Though we have discussed evidence of symbiont-conferred pesticide tolerance and/or direct biotransformation of pesticides by insect-associated bacteria, the amenability of these microbes to this type of manipulation and the specific pathways utilized for chemical conversion remain unknown. The potential for bioremediation is likely reliant on the cultivability of the symbionts of interest. This is not a reality for many insect-associated bacteria and, as such, the dependence on culturable, tractable organisms for bioremediation is a caveat that reduces its present feasibility and may be prohibitive.

Many of the organisms associated with pesticide tolerance have yet to be identified. Instead, reduced pesticide tolerance of a host is often the result of antibiotic treatment causing dysbiosis (Table 1). This strategy may overestimate the role individual symbionts play in conferring pesticide tolerance. Additionally, the culture-independent approaches utilized in many studies are several steps behind practical utility in bioremediation development pipelines, as described above.

However, by dedicating time and energy to identifying, characterizing, culturing, and potentially engineering these insect symbionts, they could provide a means of pesticide clean-up in saturated agricultural fields, watershed, and soils. With further development, inoculation or bioaugmentation of detoxifying bacteria could serve to reduce pesticide residuals in the environment thereby improving ecosystem health while lessening the deleterious effects of lingering pesticides. Insect-associated, pesticide-degrading bacteria have the potential for a larger impact on environmental stewardship in multiple spheres of research including, but not limited to, pest control and bioremediation.

## Closing Remarks

The impact strides in the field of insect-associated microbiota have made related to pesticide degradation should inform the methods, monitoring, and development of ecological pest management strategies. Given the diversity of insects and symbionts implicated, this mechanism of chemical detoxification by bacteria is pervasive. The arms race between us and pests must be informed by these discoveries and pest control strategies must expand to consider the multifaceted nature of these ecological interactions. Additionally, symbionts also could be the key for bioremediation of pesticides particularly when they are isolated from those same environments. Environmental clean-up efforts could potentially use these identified organisms to help guide their research, development and deployment plans for remediating natural settings. In the end, the key to stewardship of chemical pesticide is and always has been environmental awareness. This includes monitoring the responses sustained use triggers in the environment not only in insect pest species, but their microbial partners.

## Author Contributions

AB and BP developed, wrote, and revised the ideas and content presented in this manuscript. Both authors approved the publishing of this manuscript and take responsibility for all of its contents.

## Conflict of Interest

The authors declare that the research was conducted in the absence of any commercial or financial relationships that could be construed as a potential conflict of interest.
